# Management and outcome of patients with cardiac arrest after avalanche accidents in the Swiss Alps: A retrospective analysis

**DOI:** 10.1016/j.resplu.2025.100922

**Published:** 2025-03-04

**Authors:** Jürgen Knapp, Daniel Höftmann, Roland Albrecht, Sven Straumann, Mathieu Pasquier, Urs Pietsch

**Affiliations:** a Department of Anaesthesiology and Pain Medicine, Bern University Hospital, Inselspital, University of Bern, Bern, Switzerland; b Swiss Air-Rescue (Rega), Zurich, Switzerland; c Department of Anaesthesiology and Intensive Care Medicine, Cantonal Hospital Münsterlingen, Münsterlingen, Switzerland; d Department of Emergency Medicine, Bern University Hospital, Inselspital, University of Bern, Bern, Switzerland; e Department of Intensive Care Medicine, Cantonal Hospital St. Gallen, St. Gallen, Switzerland; f Department of Intensive Care, Amsterdam University Medical Center, Amsterdam, the Netherlands; g Department of Emergency Medicine, Lausanne University Hospital and University of Lausanne, Lausanne, Switzerland

**Keywords:** Asphyxia, Avalanche, Cardiopulmonary resuscitation, Extracorporeal life support, Outcome, Helicopter emergency medical service, Hypothermia, Accidental

## Abstract

**Aim:**

Our aim is to evaluate the management and outcome of avalanche victims in cardiac arrest (CA), focusing on the adherence to international management guidelines and to identify ways to improve the future care of avalanche victims through retrospective evaluation of the missions.

**Methods:**

We analysed a retrospective cohort of all avalanche victims in CA treated by Swiss Air-Rescue Rega between 2010 and 2024. Data regarding the avalanche burial (type of burial, burial duration, presence of a patent airway) were evaluated, as were helicopter operational data, data on prehospital medical care [cardiopulmonary resuscitation (CPR) efforts, airway management, core temperature], transport destination, data from further in-hospital treatment if applicable [core temperature, type of rewarming, serum potassium levels, extracorporeal life support (ECLS)] as well as patient outcome.

**Results:**

147 patients could be evaluated. 50 (34%) were declared dead without CPR efforts. CPR was started in 97 patients (66%), of whom 19 achieved ROSC (13%). Only 4 of these patients survived to hospital discharge (3%), 3 of whom had a good neurological outcome (2%). 34 patients (23%) were transported to hospital while CPR was ongoing, of whom in 11 (7%) ECLS was tried to initiate. None of these patients survived to hospital discharge. 27 patients (18%) were not treated in accordance with the guidelines. 22 of these (15%) were (potentially) undertreated (mainly in the sense of transport to a non-ECLS centre, although an ECLS centre would have been correct), 5 (3%) were overtreated (mainly in the sense of transport under ongoing CPR, although not indicated). 61% were tracheally intubated. On admission, core temperature was 1.9 °C (95% confidence interval 1.1–2.7) lower than the temperature measured on scene.

**Conclusions:**

Patients who suffer a CA in avalanche accidents have a very poor outcome. A high proportion of patients were not tracheally intubated during transport, cooled down further during resuscitation and transport or were not transported to ECLS centres although indicated. On the other hand, the outcome of ECLS patients is extremely poor.

## Introduction

Every year more than 100 people die in avalanches in Europe, in Switzerland alone about 25.^1^, ^2^ Despite ever-improving prevention measures, better rescue technology, reconnaissance campaigns and an increasingly dense network of rescue helicopters, this figure has remained constant in recent decades.^3^ Avalanche victims are often relatively young and healthy recreational athletes, resulting in a high loss of potential years of life compared to typical patients suffering an out-of-hospital cardiac arrest (OHCA).^4^

The pre-hospital management of avalanche victims in cardiac arrest is challenging, such rescue missions typically occurring in winter and often under unfavourable weather conditions. Decisions on the initiation or termination of cardiopulmonary resuscitation measures (CPR) or the transport of patients under ongoing CPR often have to be made under time pressure and with limited information. A corresponding guideline from the Medical Commission of the International Commission of Alpine Rescue (ICAR MEDCOM) to support rescue personnel with decision making in this regard was incorporated into the European Resuscitation Council (ERC) guidelines in 2015 and has been regularly updated since.^5^, ^6^, ^7^

The aim of the current study is to evaluate the management and outcome of avalanche victims in cardiac arrest, focusing on the adherence to international management guidelines. In addition, we are trying to identify ways to improve the future care of avalanche victims through retrospective evaluation of the missions.

## Methods

Swiss Air-Rescue (Rega) operates 14 helicopter bases, which are distributed throughout the country covering an area of more than 40,000 km^2^, and conducts on average 50 helicopter emergency medical service (HEMS) missions per year for avalanche accidents, and thus about 30% of all avalanche accidents in the European Alps.^3^ We reviewed all rescue missions conducted for avalanche accidents between January 2010 and May 2024 by Rega. We included all avalanche victims with OHCA, regardless whether they declared dead on scene or transported to a hospital. The following data were extracted from each patient’s pre-hospital chart: age, sex, National Advisory Committee for Aeronautics (NACA)-score, location, operational data, destination hospital, type of burial and burial time (critical burial was defined as head and chest buried under snow), presence of an air pocket, type of airway management, core temperature on scene, suspected lethal trauma, provision and outcome of pre-hospital CPR efforts [terminated on scene, restoration of spontaneous circulation (ROSC) on scene or transport to hospital under CPR].

We obtained the following data for patients transported to hospitals: core temperature and blood potassium levels at hospital admission, presence and type of traumatic injuries, and method of rewarming. Outcome parameters derived from hospital charts consisted of: survival to hospital discharge and neurological outcome for survivors using the cerebral performance category (CPC).^8^ A good neurological outcome was defined as CPC 1 or 2.

To analyse guideline adherence, we assessed whether the management of each individual patient followed the recommendations of the international guidelines for the resuscitation of avalanche victims as defined by ICAR MEDCOM and the ERC (at the corresponding point in time of the rescue operation).^5^, ^6^ Under-treatment was assumed if measures that would have been recommended according to the guidelines were not carried out or were discontinued prematurely, or if a patient was not transported to an ECLS centre despite such recommendation in the guidelines. Conversely, over-treatment was defined as measures or transport decisions that are not actually recommended by the guidelines.

### Statistical analysis

The collected data were entered into an Excel spreadsheet (Microsoft, Redmond, WA, USA). In terms of summary statistics, categorical variables are presented with counts and percentages. Continuous variables are presented as median and interquartile range (IQR).

### Ethics

The study was conducted in accordance with ethical standards as laid down in the Declaration of Helsinki and all national guidelines. The local ethics committees of St. Gallen (EKOS) approved the study and granted permission to use patient data without individual informed consent according to the federal act on research involving human beings and the ordinance on human research with the exception of clinical trials. The permission also covered the use of patient data regarding HEMS operations (reference number 2024-00511; EKOS 24/041).

## Results

A total of 159 victims with OHCA were identified, out of to a total number of 129 avalanches. The documentation was incomplete for 12 patients, leaving 147 cases for analysis. Six (4%) avalanche victims were partially and 141 (96%) critically buried. Median age of the patients was 41 years (IQR 31–52), 90% were male.

### Overall outcomes

**Fig. 1:**
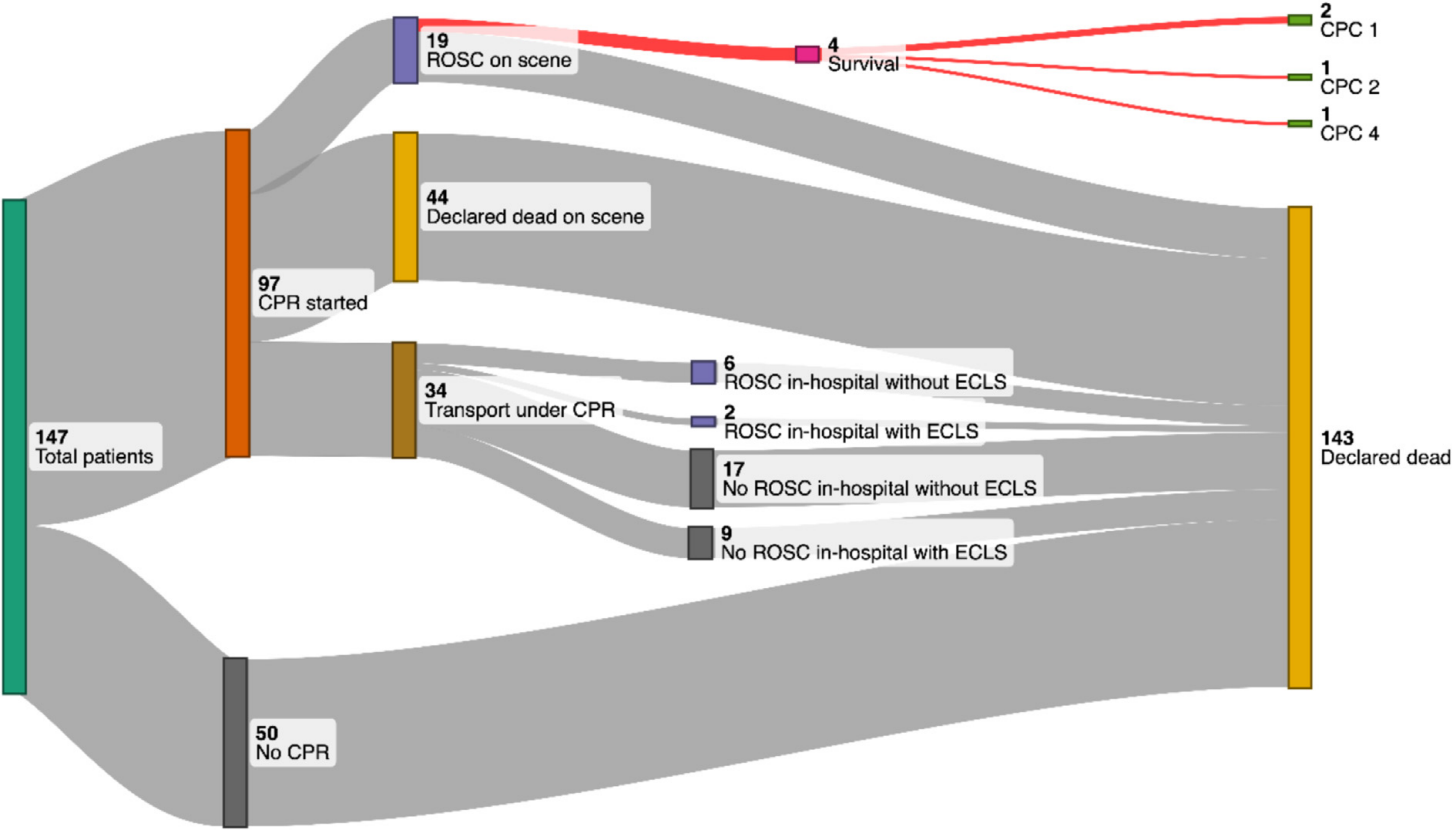
Sankey diagram of the management and outcome of patients with out-of-hospital cardiac arrest after an avalanche accident. CPC: cerebral performance category. CPR: cardiopulmonary resuscitation. ECLS: extracorporeal life support. ROSC: restoration of spontaneous circulation.

The management and outcome of the 147 cases is summarised in Fig. 1. CPR was not initiated in 50 patients (34%) because the patient’s body was completely frozen (*n* = 11), a lethal trauma was suspected (*n* = 22), or obvious signs of death were identified (*n* = 17).

CPR was initiated in 97 patients (66%). In 44 of these (30%) CPR was stopped on scene, because of failure to achieve ROSC (*n* = 42) or because certain signs of death were only detected after CPR had already been started (*n* = 2). 34 patients (23%) were transported under ongoing CPR. None of these 34 patients survived to hospital discharge. In 19 patients on which CPR was started (13%) sustained ROSC could be achieved on scene. The median burial time of these patients was 30 min (IQR 22.5–30) with a minimum of 0 min (partial burial) and a maximum of 68 min (this patient died one day after the accident from multiple organ failure). A favourable neurological outcome was achieved in three patients (2% of the total, 3% of all patients in whom CPR was initiated). Details on these four surviving patients can be found in Table 1.

**Table 1:** Characteristics of patients with out-of-hospital cardiac arrest after an avalanche accident who survived to hospital discharge.

Age (years), sex	Burial time and type of burial (min)	Air pocket	ROSC on scene	Temperature (°C)	CPC	Diagnosis
7, F	10 min, critical burial, roof avalanche	No	Yes	31.7	1	Aspiration pneumonia, no evidence of hypoxic neurological damage, mild coagulation disorder, hypotension, hyperglycaemia, hypokalaemia, hypothermia, no traumatic injuries
15, M	17 min, partial burial (head free), roof avalanche	Head free	Yes	33.3	1	Asphyxia in chest trauma, fractured ribs 9 and 10 on the right side, aspiration, no signs of hypoxic brain damage, delirium
21, M	35 min, critical burial, alpine avalanche	No	Yes	31.5	2	Hypoxic brain damage, short term diabetes insipidus centralis, status epilepticus, aspiration pneumonia, acute kidney injury AKIN 2, mild coagulation disorder, no traumatic injuries
55, M	35 min, critical burial, alpine avalanche	Unknown	Yes	33.1	4	Severe hypoxic encephalopathy

CPC: cerebral performance category.

F: female.

M: male.

ROSC: restoration of spontaneous circulation.

### In-hospital outcomes

53 patients (36%) received further in-hospital care, either after having achieved ROSC on scene or under ongoing CPR measures. In 16 patients (11%) CPR was stopped at hospital admission without further rewarming measures. In five patients (3%) rewarming measures were started: two cases with external non-invasive rewarming measures (Bear Hugger® or Arctic Sun®), three cases with intravascular rewarming (Coolguard®). For two cases we could not obtain information regarding intra-hospital rewarming measures. None of these patients achieved ROSC and CPR was discontinued. In all of these cases the decision to discontinue resuscitation measures was made due to one or several of the following reasons: a long burial time, a long duration of CPR and/or serum potassium levels >10 mmol/L.

Extracorporeal life support (ECLS) rewarming either by cardiopulmonary bypass or by veno-arterial extracorporeal membrane oxygenation was attempted in 11 patients (7%). Cannulation failed in 3 patients: one due to exsanguination, one due to blood clotting in the large vessels, and for one due to undocumented reasons. None of the eight patients in whom ECLS could be started (5% of all avalanche victims) survived to hospital discharge. The decision to discontinue ECLS and intensive care treatment was made due to one or several of the following reasons: uncontrollable bleeding complications (*n* = 6), inability to achieve sufficient cardiac output (*n* = 4), severe hypoxic brain damage or lack of flow signal in the Doppler examination of the cerebral arteries (*n* = 4), capillary leak syndrome (*n* = 3), abdominal compartment syndrome (*n* = 1) and/or multiple organ failure (*n* = 1). Table 2 provides an overview of all patients in whom ECLS therapy was attempted. Characteristics of patients transported to hospital under ongoing CPR without an attempt of ECLS is shown in Table 3.

**Table 2:** Characteristics of patients with out-of-hospital cardiac arrest after an avalanche accident and for whom extracorporeal life support (ECLS) was attempted to initiate at hospital, clinical problems and reasoning for discontinuation of ECLS or cannulation attempts.

Age (years), sex	Airway patency	Temperature (°C)	Serum potassium (mmol/L)	Burial duration (min)	Duration of CPR to hospital admission (min)	HOPE Score	Total duration of CPR (min)	Clinical situation and diagnoses	ECLS implementation successful
33, M	Patent	30.1	4.7	30	55	14	n.a.	Uncontrollable coagulation disorder under ECLS	yes
40, M	Patent	29	6.9	33	60	7	90	Core temperature raised to 34 °C via ECLS, no traumatic events in imaging, deranged coagulation, massive transfusion of blood products, various bleedings from injection sites and during bronchoscopy, no further atelectasis, and abdominal compartment without visible bleeding. Transcranial Doppler proofing no blood flow in both hemispheres. Decision to discontinue therapy due to probably lethal cerebral edema.	yes
31, M	Patent	29	6.0	42	46	14	73	Discontinuation of ECLS, termination criteria: failure to regain circulation, multiple organ failure	yes
27, M	Not patent	28.2	16	90	60	0	85	During arterial cannulation for ECLS the aorta was seen to be filled up with clotted blood to a large extend. Cancellation of further cannulation.	no
27, M	Patent	27	3.5	75	60	63	n.a.	Not able to establish sufficient circulation with ECLS, substitution of various blood products and coagulation factor concentrates. CT: multiple serial rib fractures, multiple spinal fractures, CCT: uncus herniation.	yes
52, M	Not patent	30	n.a.	57	35	n.a.	55	Abortion of ECLS cannulation as large arteries completely collapsed, TEE shows empty ventricles, with distended abdomen, most likely exsanguation.	no
37, M	Patent	21.4	7.5	45	90	31	125	Midface fracture right Le-Fort III, left II, rib series fracture on both sides, small bilateral pneumothoraces, severe TBI, severe hypoxic brain damage, abdominal compartment, coagulation problems, bulbar bursting, initially good cardiac ejection under ECLS, then deterioration in abdominal compartment. Laparotomy proofed no bleeding, capillary leak, disseminated intravascular coagulation was controlled. In follow-up CCT: increasing signs of irreversible hypoxic brain damage.	yes
36, M	Patent	22.1	5.1	10	95	55	115	Massive transfusions required during ECLS, massively distended abdomen with progressive free fluid, spontaneous bleeding at the nose, bladder catheter and scrotal hematoma. Increasing bruising on the torso, haemoglobin concentration never above 64 g/L despite massive transfusion, pH finally 6.8. Decision to discontinue ECLS without laparotomy 44 min after admission.	yes
23, M	Not patent	25.7	11.8	120	75	1	123	Pneumothorax on the right side, little free fluid in the small pelvis. Attempted cannulation for ECLS unsuccessful despite of massive venous volume administration. Lots of blood from the tracheal tube most likely due to hypothermia and coagulation disorder at 26.5 °C.	no
31, M	Patent	23.6	9.5	Several hours	21	36	164	After reaching 31.7 °C core temperature, stagnation of rewarming, anuria, hyperkalemia, pulmonary edema, sharp decrease in cerebral oximetry. No signs of myocardial activity in TEE. Therefore, decision to terminate resuscitation measures.	yes
56, M	Not patent	24.6	9.6	35	114	1	139	CT shows signs of hypoxic brain damage, small pneumothorax, sternal fracture, rib fracture, suspected renal infarction, pancreatic contusion, no cardiac action on ultrasound.	yes

Total duration of CPR: Duration of cardiopulmonary resuscitation from the start at the scene of the accident until ECLS is started or cannulation attempts are abandoned.

CCT: cerebral computer tomography.

CPR: cardiopulmonary resuscitation.

CT: computer tomography.

F: female.

HOPE Score: Hypothermia Outcome Prediction after ECLS Score.

M: male.

n.a.: not available.

TEE: transesophageal echocardiography.

**Table 3:** Characteristics of patients of patients with out-of-hospital cardiac arrest after an avalanche accident who were transported to hospital under ongoing cardiopulmonary resuscitation but without attempts of extracorporeal life support.

Age (years), sex	Airway patency	Temperature (°C)	Serum potassium (mmol/L)	Burial duration (min)	HOPE Score	Total duration of CPR (min)
48, M	Unclear	28.8	7.6	90–120	8	296
39, M	Patent	25	12	60	6	110
61, M	Patent	28	11.5	30	2	78
36, M	Patent	Temperature probe malfunction	9.3	53	n.a.	59
14, M	Patent	31.3	5.0	45	12	n.a.
49, F	Patent	26.0	3.6	45	85	93
38, M	Unclear	33.2	n.a.	80	n.a.	43
52, M	Patent	30.2	5.1	Unclear	16	70
32, M	Patent	33.9	10.8	60	0	48
39, M	Patent	22.0	9.7	60	24	57
59, M	Patent	27.6	6.5	35	6	120
50, M	Unclear	32.2	6.1	30	1	159
58, M	Unclear	31	14.2	80	0	95
32, F	Patent	n.a.	7	40	n.a.	68
40, F	Unclear	30	6.7	70	18	76
31, M	Not patent	30.0	3.3	30	6	70
43, F	Not patent	n.a.	n.a.	15	n.a.	79
55, M	Not patent	24.8	12.2	180	1	90
82, M	Not patent	n.a.	n.a.	35	n.a.	78
19, M	Patent	29.1	13.7	54	1	136
56, M	Patent	n.a.	22	Unclear	n.a.	32
55, F	Unclear	24.9	15.2	140	10	78
41, M	Patent	29.3	7.6	44	11	74

Total duration of CPR: Duration of cardiopulmonary resuscitation from the start at the scene of the accident until termination of resuscitation measures.

CPR: cardiopulmonary resuscitation.

F: female.

HOPE Score: Hypothermia Outcome Prediction after ECLS Score.

M: male.

n.a.: not available.

### Airway management

61% of patients in whom CPR was started or continued by the HEMS crew were intubated tracheally. A supraglottic device was used in 4% and 35% were ventilated with a bag-valve-mask. Two of the 19 patients with ROSC on scene could be transported to hospital breathing spontaneously without advanced airway management, 16 were tracheally intubated (eight of whom before ROSC, eight after ROSC) and one patient via a supraglottic airway (inserted before ROSC). Of the 34 patients who were transported under ongoing CPR, 24 were intubated, 8 were bag-mask ventilated and 2 via a supraglottic device.

### Adherence to guidelines for resuscitation in avalanche victims

In 118 patients (80%) treatment on scene was in accordance to guidelines. The characteristics of the 27 patients (20%) not treated according to the guidelines valid at the time of the accident are shown in Table 4. The most frequent deviation from the specified recommendations was an under-treatment in the sense that the patient was not transported to an ECLS centre, although current guidelines would have indicated this approach. On the other hand, five patients were transported to an ECLS centre (four primarily, one patient as a secondary transport) despite this approach not being indicated according to the guidelines that were valid at the time of the rescue operation.

**Table 4:** Cases of under-treatment (in relation to guideline recommendations at the time of the accident), potential under-treatment, and over-treatment (in relation to guideline recommendations at the time of the accident) of patients with out-of-hospital cardiac arrest after an avalanche accident.

Under-treatment (n = 20)	Number of cases
Termination of CPR instead of transport to ECLS center despite unknown relevant prognostic factors (burial time, ECG rhythm, presence of an air pocket) and no certain signs of death	7
Transport to non-ECLS center despite burial <60 min and core temperature <30 °C or unknown	6
Transfer to non-ECLS center despite cardiocirculatory instability after ROSC	5
CPR duration <20 min with temperature ≥30 °C in absence of an obstructed airway or lethal trauma	1
Transport to non-ECLS center despite witnessed cardiac arrest after rescue (“rescue collapse”)	1
**Potential under-treatment (n = 2)**	**Number of cases**

Transport to non-ECLS center with ROSC despite unknown temperature (in-hospital core body temperature 29 °C)	1
Transport to non-ECLS center although ECLS center would have been indicated according to body temperature due deviation in temperature measurement pre-hospital vs. in-hospital	1
**Over-treatment (n = 5)**	**Number of cases**

Transport under CPR without indication (asystole, burial time >60 min, core temperature ≥30 °C, and out-of-hospital CPR >20 min)	4
Secondary transport to ECLS-center under ongoing CPR despite serum potassium >8 mmol/L	1

CPR: cardiopulmonary resuscitation.

ECLS: extracorporeal life support.

ROSC: restoration of spontaneous circulation.

### Secondary transport

Two patients were transported to a regional hospital under ongoing CPR. In both cases, the patients were transported secondarily to an ECLS centre. We estimated the time delay between the actual arrival of the patients at the ECLS centre and a simulated scenario in which the patient had been transported directly to the centre based on the flight distance. This was 103 and 105 min, respectively. With regard to guideline adherence, further transport was indicated for only one of them.

### Core temperature

**Fig. 2:**
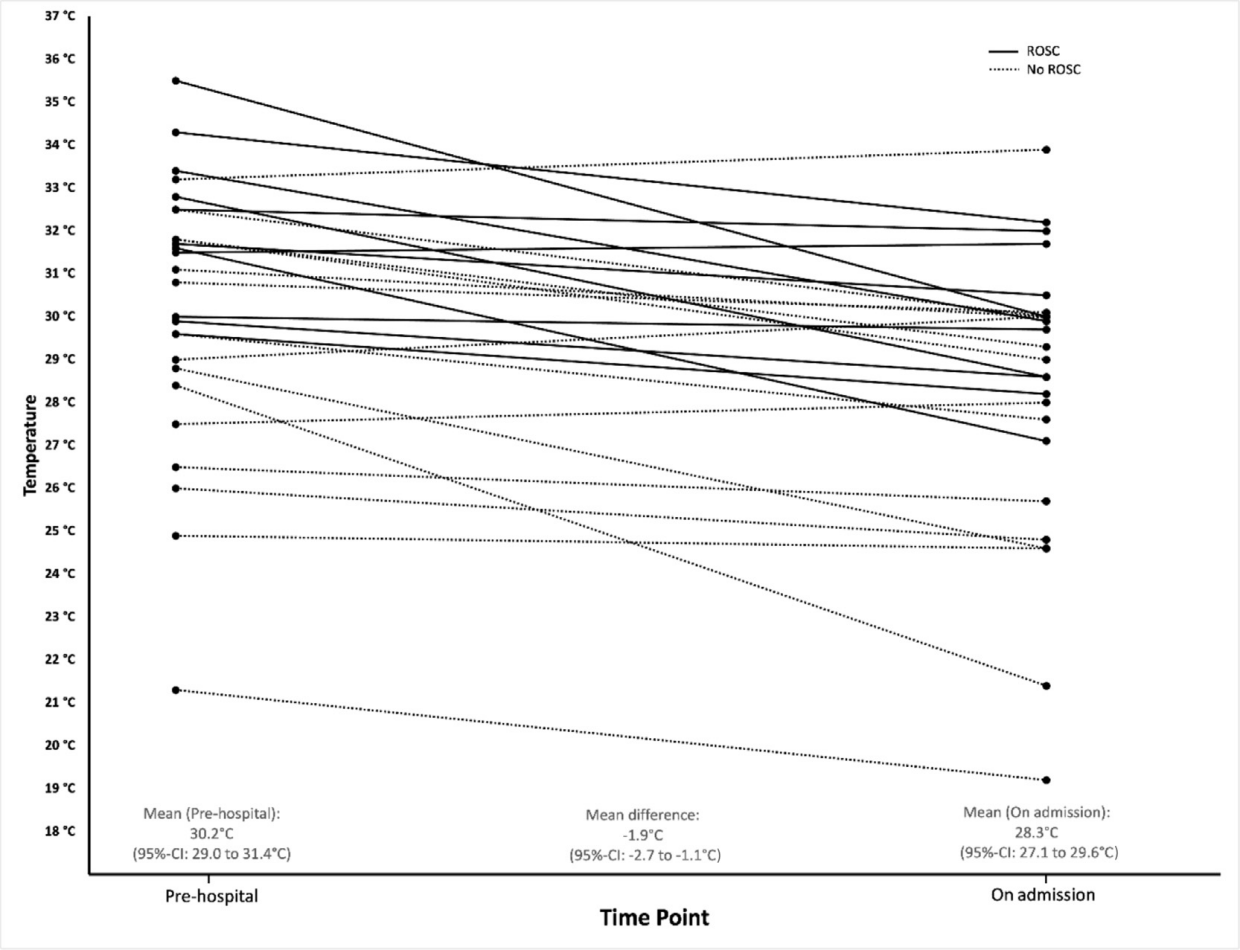
Prehospital and hospital temperatures of patients with out-of-hospital cardiac arrest after an avalanche accident, depending on the restoration of spontaneous circulation (ROSC, black line) or not (dotted line).

In 27 patients, we were able to compare the core temperature measured on scene with the core body temperature measured at hospital admission. This shows that avalanche victims cool down by an average of 1.9 °C (95% confidence interval 1.1–2.7 °C) during rescue and transport (Fig. 2). The mean rate of cooling was 0.05 °C/min in patients with ROSC and 0.03 °C/min in patients without ROSC.

### Trauma

Information about the presence of trauma was available for 27 patients. The most common injury were thoracic injuries (*n* = 15) and injuries of the abdomen (*n* = 8). Six patients had a clinical relevant pneumothorax at hospital admission. The burial times of patients with pneumothorax were between 25 and 120 min. With short burial times <30 min in one patient and long burial times of >60 min in three patients. In contrast, injuries of the cervical spine and traumatic brain injuries were only observed in two and three patients, respectively.

## Discussion

### Outcome

CPR was started in 66% of patients. Most patients (64%) were declared dead on scene. Those percentages are comparable to previous studies conducted in the European Alps.^9^, ^10^, ^11^ Our study supports previous findings that the outcome of CPR measures on avalanche victims is generally very poor. In our cohort, the chance of survival with a favourable neurological outcome after OHCA due to an avalanche accident was only 2%. This corresponds well with the findings of four previous studies conducted on avalanche accidents in the Alps. These show rates between 0 and 6.3%, with an average of 1.8%.^9^, ^10^, ^11^, ^12^

One of the three patients who survived with favourable neurological outcome was only partially buried. Such a case, in which a cardiac arrest was very likely caused by compression of the thorax by snow, has only been described once in the literature to date.^13^ The other two patients had burial times of 10 min and 35 min, respectively. Two of the survivors with favourable neurological outcome (the one partially buried and the one with a burial time of 10 min, both with CPC 1) were buried by a roof avalanche, which typically corresponds with a significantly shallower burial depth due to the smaller amount of snow compared to avalanches in open terrain. Furthermore, the area in which a patient may be buried is also much smaller for roof avalanches compared to avalanches in open terrain. Thus, we can only report one survivor with a good neurological outcome (CPC 2) after OHCA following burial in an avalanche in open terrain in the past 15 years. Even of the 50 patients with a documented open airway or air pocket, in whom hypothermic OHCA could safely be assumed, not a single patient survived who did not achieve ROSC on scene. In general, good outcome in arrested survivors seems associated with burial <60 min, no multiple trauma and ROSC on scene.

The number of patients transported to hospital under ongoing CPR corresponds approximately to the observation of other studies with an average of 28%.^11^ However, in our patient population, this must partially be considered as ‘over-treatment’ since this was not in line with guidelines in four of the patients (12% of all patients transported under CPR) which is considerably higher than the 5% reported by Metrailler et al.^11^

On the other side, we also identified cases of under-treatment, specifically patients who were not transported for ECLS rewarming despite an indication to do so according to current guidelines. The detection of these patients is critical, as survivors of OHCA following an avalanche accident who had a long (>60 min) burial duration have excellent neurological outcomes, since hypothermia is the most likely cause of cardiac arrest (CA). However, our findings are in line with other studies, who showed potential for improvement in the detection of hypothermic CA patients following a long burial.^9^, ^11^, ^14^ According to the current guidelines, unless the body is completely frozen, there is obvious lethal trauma, or the initial rhythm is asystole without a patent airways, avalanche victims in OHCA after a long burial (>60 min) should be considered to be in hypothermic CA and hence be transported under CPR to a hospital capable of ECLS.^14^, ^15^

Our results are also rather disappointing with regard to the benefits of ECLS. Of the 11 patients treated with ECLS, none survived. This is also consistent with the results of previous studies.^9^, ^10^, ^11^, ^12^ In these, an average of about 1% of patients treated with ECLS survived. However, in all four patients that were reported with successful ECLS in the studies by Strapazzon et al.^9^ and Boué et al.^12^, the OHCA was due to a rescue collapse (i.e. after the uncovering of the patients due to central shift of peripheral cold blood). We believe data on the usefulness of ECLS in OHCA due to an avalanche accident is not yet sufficient to draw definite conclusions. However, our and the previously reported data would support the notion that the benefit of ECLS for avalanche victims who are already in CA when they are found is widely overestimated, since only 1% of avalanche victims suffer from hypothermic circulatory arrest.^16^

An internationally established score for estimating the probability of survival in hypothermic cardiac arrest patients undergoing ECLS is the Hypothermia Outcome Prediction after ECLS (HOPE) score.^6^, ^17^, ^18^ We were able to retrospectively calculate the HOPE-score for 10 of our 11 ECLS patients. In four of these patients the HOPE-score was <10, indicating a survival probability of <10% in these patients. This supports other studies in which the use of the HOPE score has been showed to reduce over-treatment (and therefore futile rewarming attempts), while still indicating ELCS for patients with a potential for good outcome.^17^, ^18^, ^19^ On one hand, this accounts for the extremely poor outcome of the ECLS patients in our study, and on the other hand it shows that this resource-intensive procedure may be employed too generously in avalanche victims.

### Airway

Our data, in line with the results of previous studies discussed above,^9^, ^10^, ^11^, ^12^ show that survival after OHCA in avalanche victims is based on successful on-scene treatment and chances of survival seem very much dependent on ROSC being achieved at the scene of the accident. This, together with the underlying pathophysiology of a mainly asphyxia-induced OHCA in avalanche victims^16^, emphasises the importance of sufficient ventilation. This is also reflected in the current guidelines for CPR after an avalanche accident, so that five initial ventilations (“rescue breaths”) are recommended at the beginning of CPR.^6^ In 10 of the 19 patients in whom ROSC on scene was achieved, this was achieved under bag-mask-ventilation proofing sufficient ventilation without advanced airway management in these cases. However, the high proportion of patients transported under ongoing CPR without tracheal intubation must be viewed critically since tracheal intubation appears to be advantageous with regard to ventilation in prolonged resuscitation.^20^, ^21^ On the other hand, it must be considered that poor weather conditions or a high risk of secondary avalanches could have been the reason for not performing advanced airway management on scene. In such a case, the benefit of intubation at a safe stopover or an in-cabin intubation during the flight should be considered.^22^, ^23^

### Guideline adherence

Most cases we analysed were treated in accordance with current guidelines. However, we did find major discrepancies in certain cases, especially regarding the decision to transport to an ECLS centre. 27 patients of our sample were incorrectly triaged, both in terms of under-triage (mainly no transport to an ECLS centre, although indicated) and over-triage (mainly transport to hospital, although not indicated).

In particular, the transport to the nearest regional hospital under ongoing CPR to decide whether further transport to an ECLS centre is appropriate (as proposed by the current ICAR algorithm) yielded no benefit in the two patients of our cohort, neither from a tactical nor from a medical point of view. Both patients were transported to an ECLS centre by a second helicopter while the avalanche mission was still ongoing. This led to estimated delays of more than 100 min before the patients arrived at the ECLS centre. Therefore, we believe this type of triage must be viewed very critically, especially in regions such as the European Alps with a dense network of available rescue helicopters. Our sample indicates that this approach may lead to considerable delays under conventional CPR measures before indicated ECLS treatment could be established. In retrospect, in these cases, it would certainly have been more sensible to transport the patient directly and to alert another helicopter to the avalanche operation.

Two of the patients in our cohort were transported under ongoing CPR despite a body temperature of >32 °C measured on scene. However it cannot be ruled out that safety factors may have played a role here, so that the situation on site was considered too risky to perform CPR on scene or that weather conditions may have forced a quick evacuation from the scene.

The ICAR MEDCOM recommends that the prediction of successful rewarming in an avalanche victim at hospital should include the estimation of the survival probability using the HOPE score.^15^, ^24^ This was incorporated in the ERC guidelines in 2021 and specifically in the avalanche guidelines in 2023.^6^, ^15^ Before this, guidelines recommended decision on ECLS rewarming to be based on serum potassium levels (<8 mmol/L).^5^ According to the guidelines that were current at the time of the accident, ECLS was only indicated in seven of the 11 patients we report on. Two patients after 2021 underwent ECLS despite a HOPE score of only 1% (and additionally a serum potassium level of well above 8 mmol/L) and one patient before 2021 despite a serum potassium level of 16 mmol/L. Thus, our findings reflect a worse guideline adherence compared to the study by Metrailler et al.^11^ In their study, the indication for ECLS was in line with the guidelines in all seven patients. Alas, as in our study, without any survivors. The local implementation of checklists (e.g. the avalanche resuscitation checklist by ICAR MEDCOM),^25^ regular training in how to use them and good clinical governance (e.g. morbidity and mortality case reviews, workshops, case presentations, simulation trainings) for HEMS crews could improve compliance with the guidelines.

### Temperature

The core temperature on scene was only documented in 45% of our patients. However, this measurement is extremely important as it helps to decide on the correct transport destination. If the actual core body temperature as measured after hospital admission had already been measured in the pre-hospital setting, this would have made a decisive difference in the transport destination for six patients in our study who would have been transported to an ECLS centre instead of a non-ECLS centre.

The temperature on admission to hospital was lower than that measured in the pre-hospital setting in most patients. All patients admitted to hospital with ROSC were hypothermic and almost half of these patients had a body temperature <30 °C. This indicates the importance of maintaining body temperature and active temperature management in these patients during on-site treatment and transport as well as avoiding further cooling as much as possible. Insulation from the snow, protection from wind and moisture and an external heat supply are absolutely essential.^26^, ^27^ Interestingly, patients with ROSC cooled almost twice as fast as patients who were transported while CPR was ongoing. This is most likely explained by the reperfusion of the cold extremities after ROSC and underlines the high risk of suffering a recurrent hypothermia-related circulatory arrest of avalanche victims admitted to hospital with ROSC.^27^

Avalanche victims who do not require CPR should be rescued carefully and with as little movement as possible in order to avoid further cooling of the body's core due to cold blood being washed in from the extremities since this can cause a hypothermia-induced cardiac arrest during the rescue (i.e. *rescue collapse*). Among the patient population of this study is an impressive case of rescue collapse, illustrating that the concept of rescue collapse is not only a theoretical but a real risk to patients. This avalanche victim was awake and fully alert when rescued out of the avalanche (Glasgow Coma Scale 15). He was accompanied by a rescue specialist on foot to the waiting helicopter for evacuation. On the way to the helicopter he suffered cardiac arrest and was transported to hospital under CPR, however, could not be resuscitated.

### Trauma

Twenty-two patients (15%) were declared dead on scene because of non-survivable trauma. Trauma related mortality of avalanche victims as reported in previous publications vary greatly between 6% and 24%. While studies from the European Alps report similar rates (16%^9^ and 19%^10^), the rate in Canada is significantly higher at 24%.^28^ This could be due to the different topography (forested ski terrain in Canada) and the higher proportion of ice climbers among the avalanche victims in Canada. Both our study and the Canadian study show that severe chest injuries are very common in avalanche victims requiring CPR. We observed them in more than half of the cases in which sufficient documentation was available to us to assess this parameter. In the Canadian study, chest trauma was present in 46% of single-system trauma fatalities.^28^ However, when interpreting these figures, it should be noted that chest compressions during CPR may also be the cause of the high incidence of pneumothoraces. Nevertheless, clinicians should be aware of the high probability of a relevant pneumothorax in avalanche victims, especially after CPR. However, as thoracostomies may precipitate bleeding and complicate rewarming with ECLS,^29^, ^30^ chest decompression in avalanche victims should only be performed routinely as in trauma CPR in case of short burial times. The ICAR guidelines recommend in patients in whom a hypothermic cardiac arrest is assumed, chest decompression should only be considered in cases of clinically suspected chest trauma.^15^

### Limitations

The main limitation of our study is its retrospective design. Our analysis is based on the mission protocols and hospital charts and therefore depends on the quality and scope of the information documented there. In particular, we only had reliable and valid information on the patients' injury pattern for 27 patients.

External conditions (e.g., meteorological conditions or safety concerns) are likely to have influenced decisions regarding transport strategy or medical treatment. Furthermore, our study is based only on data from Switzerland. Therefore, our results regarding transport decisions and over-treatment cannot be transferred directly to other areas outside the European Alps (e.g. with a less dense infrastructure of rescue helicopters or ECLS centres).

## Conclusions

• Overall, the prognosis for patients who suffer a CA in an avalanche accident is very poor.
• In our patient population over nearly 15 years, not a single patient who was transported to hospital under ongoing CPR survived to hospital discharge.
• We identified several cases of under-treatment. The most common of which was transport to a non-ECLS centre, although an ECLS centre would have been indicated according to guidelines. A large proportion of patients was not intubated and continued to cool down during transport.
• Over-treatment occurred in a large proportion of patients treated with ECLS although not indicated.
• The strategy of flying patients to a regional hospital under ongoing CPR in order to assess further (ECLS-)treatment in the case of multiple casualties seems to unreasonable in the setting of the European Alps.

## CRediT authorship contribution statement

**Jürgen Knapp:** Writing – original draft, Project administration, Methodology, Formal analysis, Data curation, Conceptualization. **Daniel Höftmann:** Visualization, Formal analysis, Data curation. **Roland Albrecht:** Supervision, Conceptualization. **Sven Straumann:** Writing – review & editing. **Mathieu Pasquier:** Writing – review & editing. **Urs Pietsch:** Resources, Project administration, Methodology, Data curation.

## Declaration of competing interest

The authors declare that they have no known competing financial interests or personal relationships that could have appeared to influence the work reported in this paper.
